# Computational prediction of allergenic proteins based on multi-feature fusion

**DOI:** 10.3389/fgene.2023.1294159

**Published:** 2023-10-19

**Authors:** Bin Liu, Ziman Yang, Qing Liu, Ying Zhang, Hui Ding, Hongyan Lai, Qun Li

**Affiliations:** ^1^ Department of Anesthesiology, The Fourth People’s Hospital of Sichuan Province, Chengdu, Sichuan, China; ^2^ School of Life Science and Technology, University of Electronic Science and Technology of China, Chengdu, China; ^3^ Department of Pain, The Affiliated Traditional Chinese Medicine Hospital of Southwest Medical University, Luzhou, Sichuan, China; ^4^ Department of Anesthesiology, The Affiliated Traditional Chinese Medicine Hospital of Southwest Medical University, Luzhou, Sichuan, China; ^5^ Chongqing Key Laboratory of Big Data for Bio Intelligence, Chongqing University of Posts and Telecommunications, Chongqing, China; ^6^ Research Center of Integrated Traditional Chinese and Western Medicine, The Affiliated Traditional Chinese Medicine Hospital of Southwest Medical University, Luzhou, Sichuan, China

**Keywords:** allergenic protein, multi-feature fusion, feature selection, random forest, prediction

## Abstract

Allergy is an autoimmune disorder described as an undesirable response of the immune system to typically innocuous substance in the environment. Studies have shown that the ability of proteins to trigger allergic reactions in susceptible individuals can be evaluated by bioinformatics tools. However, developing computational methods to accurately identify new allergenic proteins remains a vital challenge. This work aims to propose a machine learning model based on multi-feature fusion for predicting allergenic proteins efficiently. Firstly, we prepared a benchmark dataset of allergenic and non-allergenic protein sequences and pretested on it with a machine-learning platform. Then, three preferable feature extraction methods, including amino acid composition (AAC), dipeptide composition (DPC) and composition of *k*-spaced amino acid pairs (CKSAAP) were chosen to extract protein sequence features. Subsequently, these features were fused and optimized by Pearson correlation coefficient (PCC) and principal component analysis (PCA). Finally, the most representative features were picked out to build the optimal predictor based on random forest (RF) algorithm. Performance evaluation results via 5-fold cross-validation showed that the final model, called iAller (https://github.com/laihongyan/iAller), could precisely distinguish allergenic proteins from non-allergenic proteins. The prediction accuracy and AUC value for validation dataset achieved 91.4% and 0.97%, respectively. This model will provide guide for users to identify more allergenic proteins.

## 1 Introduction

Allergic diseases are a group of immune-mediated inflammatory response diseases, including allergic asthma, allergic rhinitis, atopic dermatitis, food allergy. These diseases are caused by the hypersensitivity of body immune system to normally harmless environmental substances (Miescher and Vogel, 2002). With the change of worldwide environment, the incidence of allergic diseases has increased considerably in the past few years. Patients with allergic diseases often have complex clinical manifestations and a high risk of recurrence (Wang et al., 2023a). Biomedical researchers are increasingly concerned about these diseases.

Substances that can induce allergic reactions, typically proteins, are called allergens (Galli et al., 2008). Allergenic proteins for humans are often derived from aeroallergens, food allergens, personal care products and so on. Allergic reactions are generally grouped into two classes. The well-studied and common class is mediated by immunoglobulin E (IgE), which is one of the five primary human immunoglobulins. An IgE-mediated (type I hypersensitivity) allergy occurs when the body encounter allergenic proteins containing immunogenic and antigenic structures. The mechanism is that allergenic proteins enter body and drive immune cells to produce lots of allergenic protein-specific IgE antibodies. When the body re-exposure to the allergenic proteins, these IgEs will bind to them and lead to the activation of other immune cells as well as the initiation of inflammation response (Platts-Mills, 2001; Oseroff et al., 2012; Guo et al., 2023). The specific recognition and interaction for allergenic proteins is based on their sequences and structures.

The common methods used to determine protein allergenicity potential are traditional immunochemical, biochemical and immunological methods (Kimber et al., 2003; Ladics and Selgrade, 2009; Zhou et al., 2023). With the development of bioinformatics and machine learning algorithms, massive computational strategies for identifying allergenic proteins have emerged and evolved over time (Saha and Raghava, 2006; Gupta et al., 2013; Sharma et al., 2021a; Lathwal et al., 2021). Thereinto, the key idea of early reported methods is to seek sequence similarity, which is mainly based on the guidelines about evaluating the potential allergenicity of novel food proteins proposed by the United Nations Food and Agriculture Organization (FAO) and the World Health Organization (WHO). These methods, such as SDAP, Allermatch, AllerTool, AllerHunter, generally assess protein potential allergenicity by searching for similar sequences on the basis of local or global sequence alignment algorithms, such as BLAST, FASTA program, etc (Ivanciuc et al., 2003; Fiers et al., 2004; Zhang et al., 2007; Muh et al., 2009). Another class of technology involves the identification of allergen-related motifs by using motif search tool, such as MEME/MAST. Furthermore, ensemble approaches, such as proAP and AlgPred 2.0, have also been developed based on both sequence similarity and motif eliciting strategy (Soeria-Atmadja et al., 2006; Wang et al., 2013; Sharma et al., 2021b). In recent years, several feature vector-based approaches have been reported, including APPEL, AllerTOP, AllergenFP, AllerCatPro, ProAll-D (Cui et al., 2007; Dimitrov et al., 2013; Dimitrov et al., 2014; Nguyen et al., 2022; Shanthappa and Kumar, 2022). In general, they take sequence-derived compositional, evolutionary, structural and physicochemical information into consideration and achieve allergenic protein classification by using machine learning or deep learning models (Wang et al., 2021; Ao et al., 2022; Wu et al., 2023). For example, random forest (RF), support vector machine (SVM), decision tree (DT), *k*-nearest neighbors (KNN) and multilayer perceptron (MLP) were employed to establish AlgPred 2.0 on the basis of composition/evolutionary information-based features (Zhang et al., 2007). Different classification models, including Gaussian Naive Bayes, Radius Neighbour’s Classifier, Bagging Classifier, ADA Boost, Linear Discriminant Analysis, Quadratic Discriminant Analysis, Extra Tree Classifier and Long Short-Term Memory (LSTM), have been considered in the study of ProAll-D (Cui et al., 2007).

**FIGURE 1:**
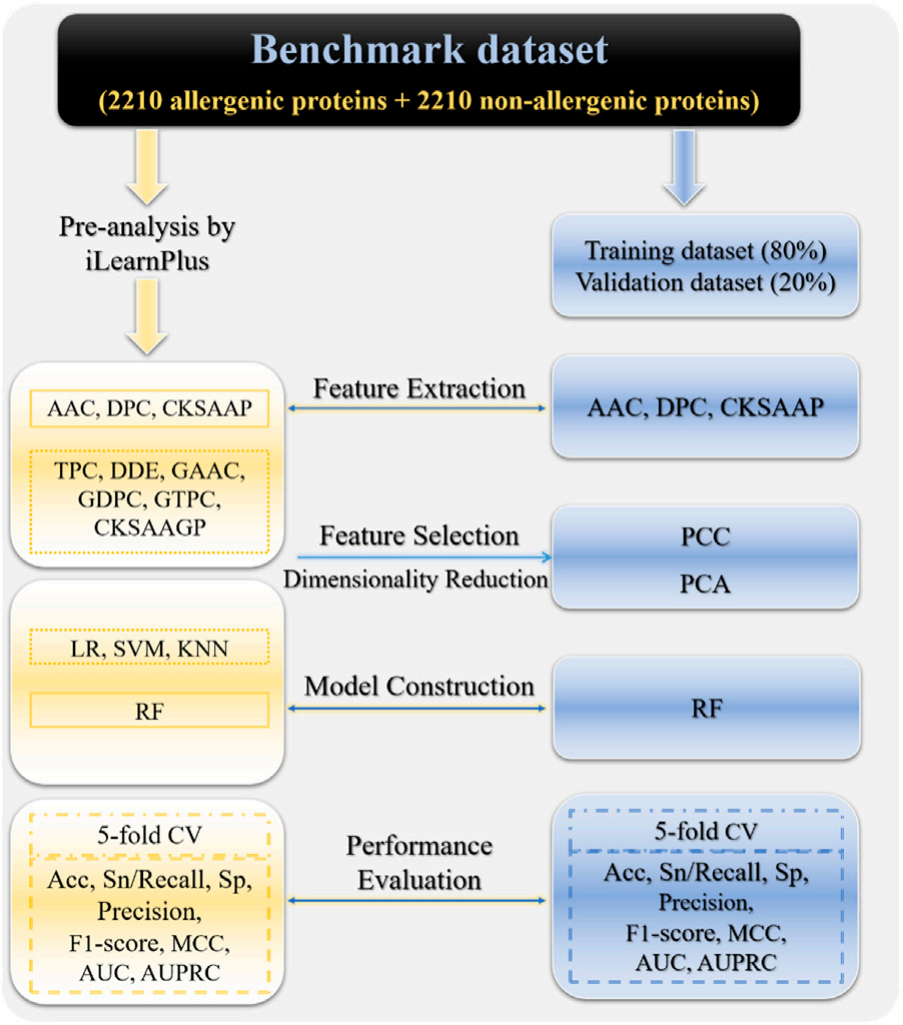
The frame of establishing the allergenic protein classifier in this study. Abbreviations: AAC, amino acid composition; DPC, dipeptide composition; TPC, tripeptides composition; DDE, dipeptide deviation from expected mean, CKSAAP, composition of *k*-spaced amino acid pairs; GAAC, grouped amino acid composition; GDPC, grouped dipeptide composition; GTPC, grouped tripeptide composition; CKSAAGP, composition of *k*-spaced amino acid group pairs; RF, random forest; LR, logistic regression; SVM, support vector machine; KNN, *k*-nearest neighbors, Acc, accuracy; Sn, sensitivity; Sp, specificity; MCC, Matthews correlation coefficient; AUC, area under receiver operating characteristic (ROC) curve; AUPRC, area under precision-recall curve.

Although there are presently a number of computational methods for detecting allergenic proteins, due to the limitations of prediction performance, it is still need to train more effective and robust allergenic protein classifiers. In this work, we focused on allergenic proteins for human beings and developed iAller to distinguish them from non-allergenic proteins. The major implement procedures have been shown in Figure 1, which includes 1) constructing a benchmark dataset consisting of 2,210 positive and 2,210 negative sample sequences; 2) conducting pre-analysis on the whole dataset with iLearnPlus by combining nine feature descriptors with four machine learning algorithms; 3) selecting AAC, DPC and CKSAAP feature extraction methods with excellent performance to encode sequence samples, as well as RF algorithm to build classifier; 4) integrating these three types of features and performing feature selection and dimensionality reduction by using PCC and PCA; 5) training and determining the optimal classifier on training dataset through 5-fold cross-validation; 6) assessing the prediction performance of the optimal RF model on validation dataset. The high accuracy and AUC value of 91.4% and 0.97 suggest that this model should be an excellent choice for identifying allergenic proteins.

## 2 Materials and methods

### 2.1 Protein sequence benchmark dataset

The main significance of this work is to provide a theoretical basis for the study of human allergic reactions. Hence, this work primarily focused on proteins causing allergic reactions in human beings, excluding other species. The sequence benchmark dataset was composed of 2,210 non-allergenic proteins and 2,210 allergenic proteins. These allergenic proteins were originated from various human allergens, mainly including wheat, rice, seafood product, pollen, dust and so on. This dataset had been studied by ProAll-D project and could be freely accessible from https://doi.org/10.17632/tjmt97xpjf.1. Proteins with high homology had been removed by CD-HIT program for avoiding sequence redundancy and ensuring the objectivity of experimental results (Shanthappa and Kumar, 2022).

### 2.2 Preliminary selection of analysis methods

For building a machine learning classifier for protein sequences, it is necessary to convert biological sequence information into feature vector information that can be processed by computers (Lu et al., 2022; Wang et al., 2023b; Dao et al., 2023; Le, 2023; Zhu et al., 2023). Therefore, it is very imortant to select appropriate feature extraction methods. For the above protein sequence datasets, we firstly performed pre-experiment by using iLearnPlus (Chen et al., 2021), a comprehensive and automated machine-learning platform. This online server could automatically generate and save evaluation metrics of the selected algorithms according to input data and parameter settings. Nine feature descriptors, including AAC, DPC, tripeptides composition (TPC), dipeptide deviation from expected mean (DDE), CKSAAP, grouped amino acid composition (GAAC), grouped dipeptide composition (GDPC), grouped tripeptide composition (GTPC) and composition of *k*-spaced amino acid group pairs (CKSAAGP), were applied to extract sample information of allergenic and non-allergenic proteins in our work. By comparing the performance of RF, logistic regression (LR), SVM and KNN classification models using these features, we finally chose the AAC, DPC, CKSAAP methods combined with RF algorithm for further detailed analysis.

### 2.3 Protein sequence features

#### 2.3.1 Amino acid composition (AAC) feature

Amino acids are the basic units of proteins. Twenty types of amino acids are involved in protein composition, namely, Alanine (A), Cysteine (C), Aspartic acid (D), Glutamic (E), Phenylalanine (F), Glycine (G), Histidine (H), Isoleucine (I), Lysine (K), Leucine (L), Methionine (M), Asparagine (N), Proline (P), Glutamine (Q), Arginine (R), Serine (S), Threonine (T), Valine (V), Tryptophan (W), Tyrosine (Y). Among the numerous computational methods for transforming protein sequences into feature vectors, AAC coding method is the simplest and most intuitive one. The principle is to calculate the frequencies of twenty types of amino acids in protein sequence (Bhasin and Raghava, 2004; Sahoo et al., 2021). Based on AAC, every allergenic/non-allergenic protein sequence can be represented with a 20-dimension feature vector, as Formula (1),

$$V_{1}={\left[f_{1}f_{2}f_{3}\;\ldots \;f_{20}\right]}^{T}$$ (1)

$$f_{i}=\frac{A_{i}}{L}$$ (2)

where T means the transpose of a vector. $A_{i}$ is the number of *i*-type amino acid contained in the protein sequence of interest, L is the total number of amino acids in the sequence, $f_{i}$ is the proportion of corresponding amino acid in this protein.

#### 2.3.2 Dipeptide composition (DPC) feature

Feature encoding method based on *k*-mer composition is to divide protein sequences into fragments with fixed length of *k*, and calculate the frequency of each type *k*-mer fragment. Such method can capture information about amino acid composition as well as local sequence order (Ahmad et al., 2016). When *k* = 2, namely, DPC, there are 20 × 20 = 400 kinds of 2-mers. Each protein sequence will be transformed into a numerical vector with 400 features. The calculation formula is as following,

$$V_{2}={\left[F_{1}F_{2}F_{3}\;\ldots \;F_{20}\ldots \;F_{400}\right]}^{T}$$ (3)

$$F_{i}=\frac{D_{i}}{L-k+1}$$ (4)

the meaning of T is same as above. L and k indicate the length of a given protein sequence and the length of small k-mer fragments, respectively. $D_{i}$ represents the total number of dipeptide i . $F_{i}$ is the corresponding proportion.

#### 2.3.3 Composition of k-spaced amino acid pairs (CKSAAP) feature

Another popular binary encoding strategy similar to DPC is CKSAAP. The encoding scheme is to count the occurrence times of 400 amino acid pairs separated by any *k*-mer in a given protein sequence (Ju and Wang, 2020). For example, when *k* = 1, a protein will be encoded to a 400-dimensional numerical vector with each feature factor being the frequency at which any one 1-spaced amino acid pair appears. In this work, we set *k*-spaced amino acid pair to *k* = 1, 2, 3 to encode allergenic and non-allergenic protein sequences, taking into account its prediction accuracy, computational time and complexity. A total of 1200 CKSAAP features were produced, as follow:

$$V_{3}={\left[\frac{N_{AA_{1}}}{N_{1}},\ldots ,\frac{N_{YY_{1}}}{N_{1}},\ldots ,\frac{N_{AA_{k}}}{N_{k}},\ldots ,\frac{N_{YY_{k}}}{N_{k}},\ldots ,\frac{N_{AA_{3}}}{N_{3}},\ldots ,\frac{N_{YY_{3}}}{N_{3}}\right]}_{20\times 20\times 3}$$ (5)

where $N_{AA_{k}}$ represents the frequency of *k*-mer separated AA pair in a protein and $N_{k}$ corresponds to the total number of *k*-spaced amino acid pairs.

### 2.4 Feature fusion, selection and shrinkage

In the field of biomolecule sequence analysis, extracting features from a single perspective often leads to incomplete sequence information and low prediction performance of classification models. In order to improve this problem, the above AAC, DPC and CKSAAP three type features were fused together for cc. Every protein sequence of the benchmark dataset was represented as:

$$V=\left[{V_{1},V}_{2},V_{3}\right]$$ (6)

Feature vectors produced by multi-feature fusion methods were usually high-dimensional and redundant (Han et al., 2022; Yan et al., 2022; Zhao-Yue ZHANG et al., 2022; Ao et al., 2023). We further utilized two approaches, PCC combined with incremental feature selection (IFS) strategy and PCA, to select more informative features and reduce dimensionality (Karl Pearson, 1901; Stigler, 1989; Dao et al., 2018). PCC is often used to measure the strength and direction of a linear relationship between two variables. It is defined as the quotient of the covariance and standard deviation between two variables. A larger absolute value of the Pearson coefficient indicates a stronger linear relationship between the two variables. PCA is another common feature extraction and dimensionality reduction method. Its purpose is to transform a series of influence factors with correlations into a new set of mutually independent comprehensive indicators, while retaining as much information as possible on the original variables during the transformation. The core idea is to map the original high-dimensional data into a new low-dimensional space and to obtain a set of orthogonal basis vectors. PCA enables the map of raw data on this set vector to be with maximum variance and preserve the major characteristics. For example, a raw dataset with *p* variables will be converted *q* comprehensive principal components by a linear combination of optimally weighted original variables, where *q* is less than p. The detailed computation procedures of these methods are described in iLearnPlus.

### 2.5 Classifier construction with random forest (RF)

RF is an ensemble learning algorithm that combines several base learners into a strong learner by voting or averaging to improve the robustness and generalization performance (Breiman, 2001; Wei et al., 2021; Yang et al., 2021; Basith et al., 2022; Islam et al., 2022; Zhang et al., 2023a). Thus, we adopted RF algorithm to construct allergenic protein classifier. The process was as follows: 1) Random sampling: N new datasets are generated by random sampling with replacement, each of which has the same size as the original dataset. 2) Building decision trees: The CART decision tree algorithm is applied to each new dataset and builds a decision tree. Due to the characteristics of random sampling, each new dataset might only contain a part of samples and features of the original dataset, as well as the predictive ability of each tree might be different. 3) Integrating: N decision trees are combined into a strong classifier by voting or averaging. The RF strategy was involved in random sampling and random feature selection. Random sampling enables the differences among each new dataset and avoids model overfitting. Random feature selection enables the variability among decision trees and improves the generalization ability of the final model.

### 2.6 Classifier performance evaluation

For assessing machine learning models more accurately, the benchmark dataset was split into training and validation datasets with ratio of 4:1. Five-fold cross-validation was used in model training. We employed several common indexes to evaluate model performance (Hasan et al., 2022; Jeon et al., 2022; Shoombuatong et al., 2022; Thi Phan et al., 2022; Zhang et al., 2023b), including accuracy (*Acc*), sensitivity (*Sn*)/recall, specificity (*Sp*), precision, F1-score (*F1*), Matthews correlation coefficient (*MCC*), area under receiver operating characteristic (*ROC*) curve (*AUC*), area under precision-recall curve (*AUPRC*) (Su et al., 2023; Yang et al., 2023). The specific equations to calculate these measures were as follows:

$$\left\{\begin{array}{c} Acc=\frac{TP+TN}{TP+FN+TN+FP}\\ Sn=\frac{TP}{TP+FN}\\ Sp=\frac{TN}{TN+FP}\\ Precision=\frac{TP}{TP+FP}\\ F1=\frac{2\times \left(precision\times recall\right)}{precision+recall}\\ MCC=\frac{TP\times TN-FP\times FN}{\sqrt{\left(TP+FP\right)\times \left(TP+FN\right)\times \left(TN+FN\right)\times \left(TN+FP\right)}} \end{array}\right.$$

where *TP* (true positive) and *FP* (false positive) denoted the numbers of sequences correctly and incorrectly classified as allergenic proteins, respectively. TN (true negative) and FN (false negative) were the numbers of samples correctly and incorrectly classified as non-allergenic proteins, respectively. The AUC and AUPRC values ranged from 0 to 1. Their higher values implied better predictive ability of models.

## 3 Results and discussion

### 3.1 Preliminary analysis results

In order to pick out the most appropriate feature extraction and model construction methods from the existing numerous algorithms, as well as to reduce experimental complexity and workload, we performed pre-analysis on the benchmark protein sequences by using iLearnPlus tool.

**FIGURE 2:**
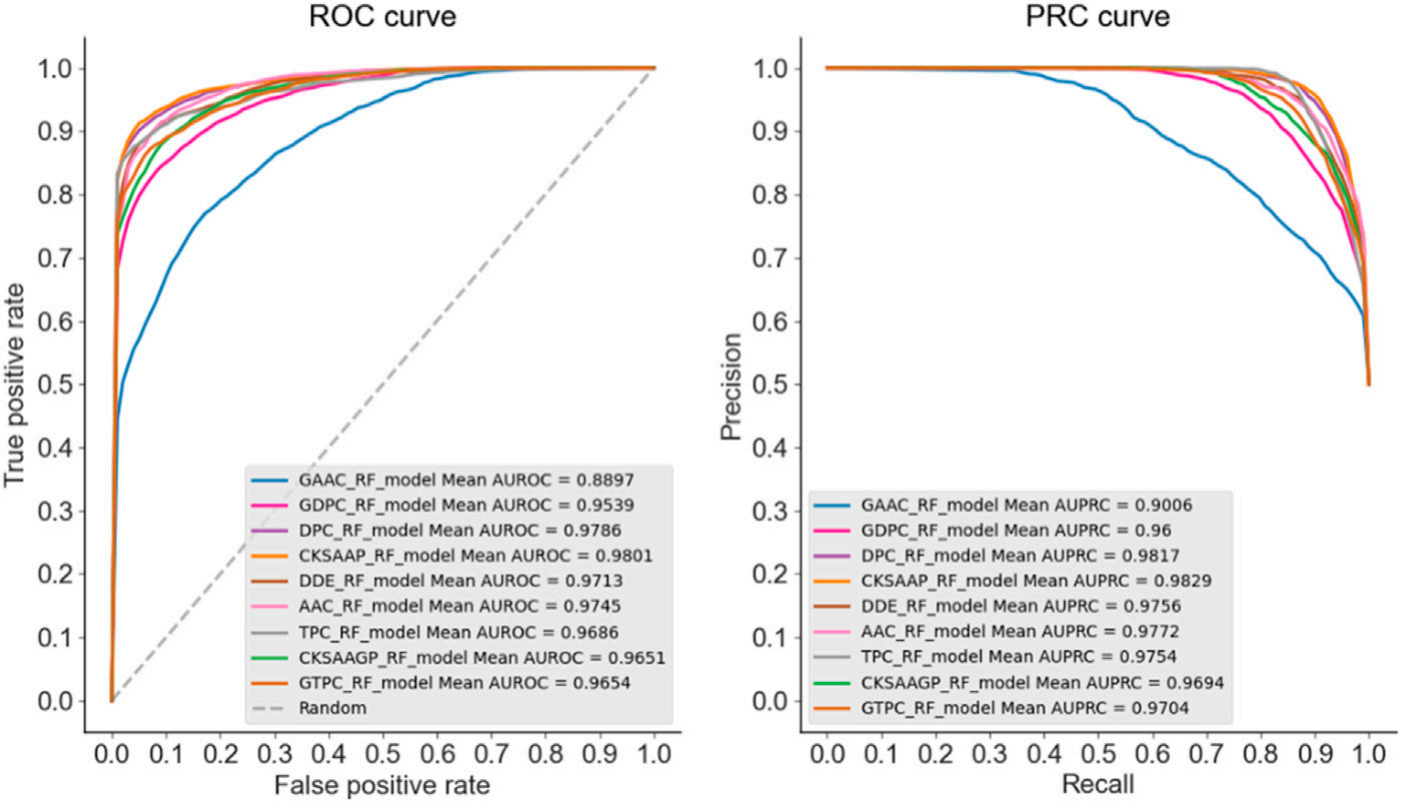
Performance of nine feature extraction methods based on RF algorithm.

In this experiment part, we firstly chosen AAC, DPC, TPC, DDE, CKSAAP, GAAC, GDPC, GTPC, CKSAAGP features to build RF, LR, SVM, KNN classification models with default parameters, respectively. The prediction performance of these nine type features were assessed by combining with RF algorithm and shown in Figure 2. It was obvious that the best-performing feature extraction methods were CKSAAP, DPC and AAC and have achieved quite high AUC and AUPRC values of about 0.98. The performance of these features based on LR, SVM, KNN algorithms (see Supplementary Figures S1–S3) also indicated that CKSAAP, DPC and AAC were more preferable methods for encoding allergenic and non-allergenic protein sequences. Secondly, we tested each of the three feature extraction methods on each machine learning classifier with corresponding optimal parameters. The best number of decision tree for RF was set as 450, the best values of *c* and *γ* for SVM were $2^{15}$ and $2^{16}$ , the *k* parameter for KNN was set as 3. The 5-fold cross-validation results on the whole benchmark dataset have been listed in Table 1. The RF classifier also outperformed other three approaches and could predict allergenic proteins accurately. Prediction accuracies and AUC values were as high as 92.0% and 0.97.

**TABLE 1:** Performance of each machine learning classifier based on AAC, DPC and CKSAAP features, respectively.

Classifier	Feature	*Sn* (%)	*Sp* (%)	*Pre* (%)	*Acc* (%)	*MCC*	*F1*	*AUC*	*AUPRC*
RF	AAC	91.9	93.0	92.9	92.4	0.85	0.92	0.97	0.98
	DPC	88.9	94.3	94.0	91.6	0.83	0.91	0.97	0.98
	CKSAAP	89.1	96.2	95.9	92.7	0.86	0.92	0.98	0.98
LR	AAC	81.0	73.1	75.1	77.0	0.54	0.78	0.85	0.84
	DPC	70.6	86.7	84.1	78.6	0.58	0.77	0.86	0.87
	CKSAAP	69.0	85.5	82.7	77.3	0.55	0.75	0.86	0.87
SVM	AAC	86.0	88.2	88.0	87.1	0.74	0.87	0.94	0.94
	DPC	83.0	85.5	85.2	84.3	0.69	0.84	0.92	0.92
	CKSAAP	83.7	85.1	84.9	84.4	0.69	0.84	0.92	0.93
KNN	AAC	89.4	87.3	87.6	88.4	0.77	0.89	0.95	0.96
	DPC	82.6	94.3	93.6	88.6	0.78	0.88	0.95	0.96
	CKSAAP	82.6	91.2	90.4	86.9	0.74	0.86	0.93	0.95

Through comprehensively comparing and analyzing the recognition performance of allergenic protein using different feature extraction methods and machine learning models, we selected AAC, DPC, CKSAAP features and RF algorithm with better performance for further detailed analysis.

### 3.2 Prediction results of multi-fusion features

To contain sequence composition information as comprehensive as possible, we fused AAC, DPC, CKSAAP features together. Each protein sequence was represented as a fused vector with 1,620 features. All the features were ranked in descending order by PCC and the top 200 features were selected out by using IFS strategy. To construct an effective and robust allergenic protein identification model, the PCA was used for further feature shrinkage. The top 100 principal features were ultimately screened out to build RF model. For determining the optimal decision tree parameter in RF algorithm, we tried to set it as 100, 200, 300, 400, 450, 500 with a cut-off value of 0.5. The prediction results (Table 2) demonstrated that the optimal value of decision tree should be set as 200, and the corresponding RF classifier could produce the highest accuracy of 91.4%.

**TABLE 2:** Prediction ability comparison of random forest models with different number of decision trees.

Number of trees	*Sn (%)*	*Sp (%)*	*Pre (%)*	*Acc (%)*	*MCC*	*F1*
100	88.9	93.0	92.7	91.0	0.82	0.91
200	88.9	93.9	93.6	91.4	0.83	0.92
300	88.5	94.1	93.8	91.3	0.83	0.91
400	88.5	93.0	92.7	90.7	0.82	0.91
450	88.8	93.7	93.3	91.2	0.83	0.91
500	88.2	93.9	93.3	91.1	0.83	0.91

**FIGURE 3:**
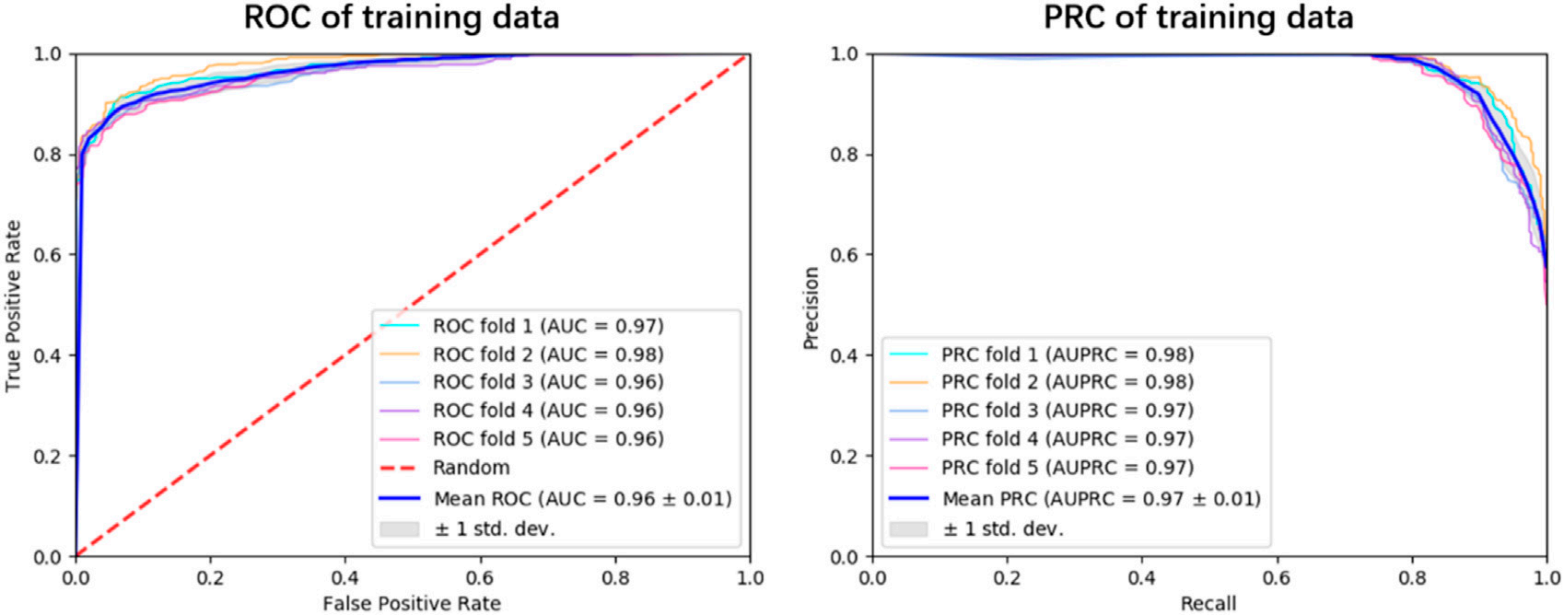
The ROC and PRC curves for the optimal RF classifier on training dataset. The blue solid line indicates the overall result of 5-fold cross-validation, the solid lines in other colors are results of each fold *k* = 1, 2, 3, 4, 5.

**FIGURE 4:**
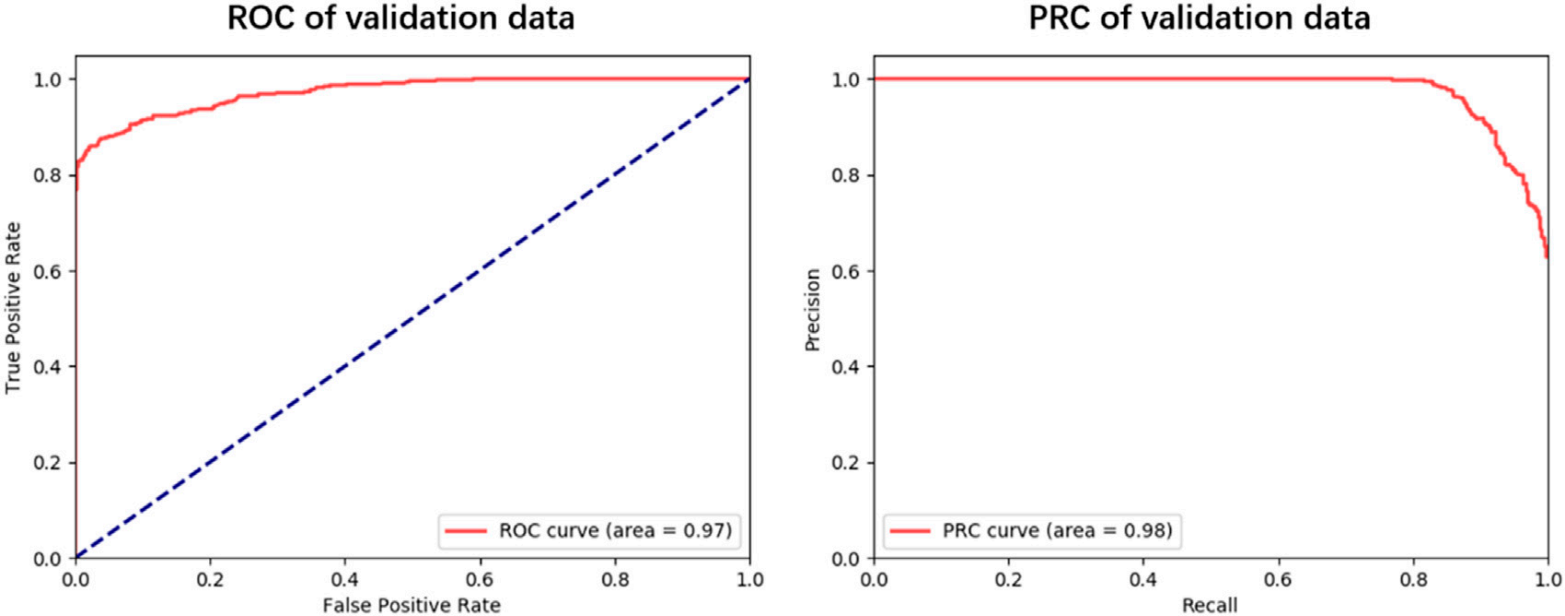
The ROC and PRC curves for the optimal RF classifier on validation dataset.

To explore the generalization ability of these fused features, we constructed the optimal RF model based on the training dataset via 5-fold cross-validation (see Figure 3; Supplementary Table S1). Allergenic protein prediction was then conducted on both training and validation datasets. Performance evaluation results were enumerated in Table 3. The RF model could produce good performance on training protein sequences. The prediction accuracy, *Sn*, *Sp*, AUC and AUPRC values were 91.0%, 88.4%, 93.6%, 0.96 and 0.97, respectively (Figure 3). In addition, it could also produce pretty good performance on the validation dataset with the accuracy of 91.4%, *Sn* of 88.9%, *Sp* of 93.9%, AUC value of 0.97 and AUPRC value of 0.98 (Figure 4). All results implied that the presented RF classifier had good performance on generalization and robustness.

**TABLE 3:** Performance summary of the optimal RF model testing on training and validation datasets by 5-fold cross-validation.

Dataset	*Sn* (%)	*Sp* (%)	*Pre (%)*	*Acc* (%)	*MCC*	*F1*	*AUC*	*AUPRC*
Training	88.4	93.6	93.3	91.0	0.83	0.91	0.96	0.97
Validation	88.9	93.9	93.6	91.4	0.83	0.91	0.97	0.98

It is quite crucial to compare with existing methods for comprehensively evaluating a novel method. Therefore, we futher compared the prediction performance of our iAller with that of AlgPred 2.0 web server (https://webs.iiitd.edu.in/raghava/algpred2/) for testing on the same validation dataset (Zhang et al., 2007). The prediction accuracy of AlgPred 2.0 with setting Machine Learning Techique as “Hybrid” was 89.8%, which was inferior to that of iAller. Moreover, AllergenFP and ProAll-D servers were established on almost the same benchmark dataset as that of this work, and the accuracy of AllergenFP was 87.9%, the best AUC value of ProAll-D was 0.92 (Cui et al., 2007; Wang et al., 2013). It implied that iAller was superior to these existing tools and could provide reliable results for researches about allergenic protein predicting.

## 4 Conclusion

Identification of allergenic proteins from the perspective of bioinformatics can provide theoretical support for the relevant biological experimental research. Although many computational models for allergenic protein prediction have been developed, few of them have been widely validated and used in related researches. Improving the accuracy and effectiveness of allergenic protein prediction remains a challenging problem. This work attempted to explore more suitable feature extraction and selection methods as well as machine learning models for identifying allergenic proteins. After a series of trials and comparative analyses, we established an effective RF model based on 100 informative fusion features via 5-fold cross-validation. The accuracy and AUC value of the classifier on validation dataset reached 91.4% and 0.97. Evaluation results suggested that this computational model, iAller, was robust and its generalization ability was superior. It indicates that fusing different types of protein sequence features is a feasible strategy. However, there is still room for improvement. In future work, more types of information such as amino acid physicochemical properties, evolutionary information will be taken into consideration, and more feature selection methods will be attempted, as well as a web server shall be constructed for bringing more convenience to researchers.

## Data Availability

The original contributions presented in the study are included in the article/Supplementary Material, further inquiries can be directed to the corresponding authors.
