# Supplementary material for: Computational prediction of allergenic proteins based on multi-feature fusion
Source: Front Genet. 2023 Oct 19;14:1294159. doi: 10.3389/fgene.2023.1294159 (PMC10622758; doi:10.3389/fgene.2023.1294159)
Supplement: Supplementary file 1 [file Table1.DOCX]

#
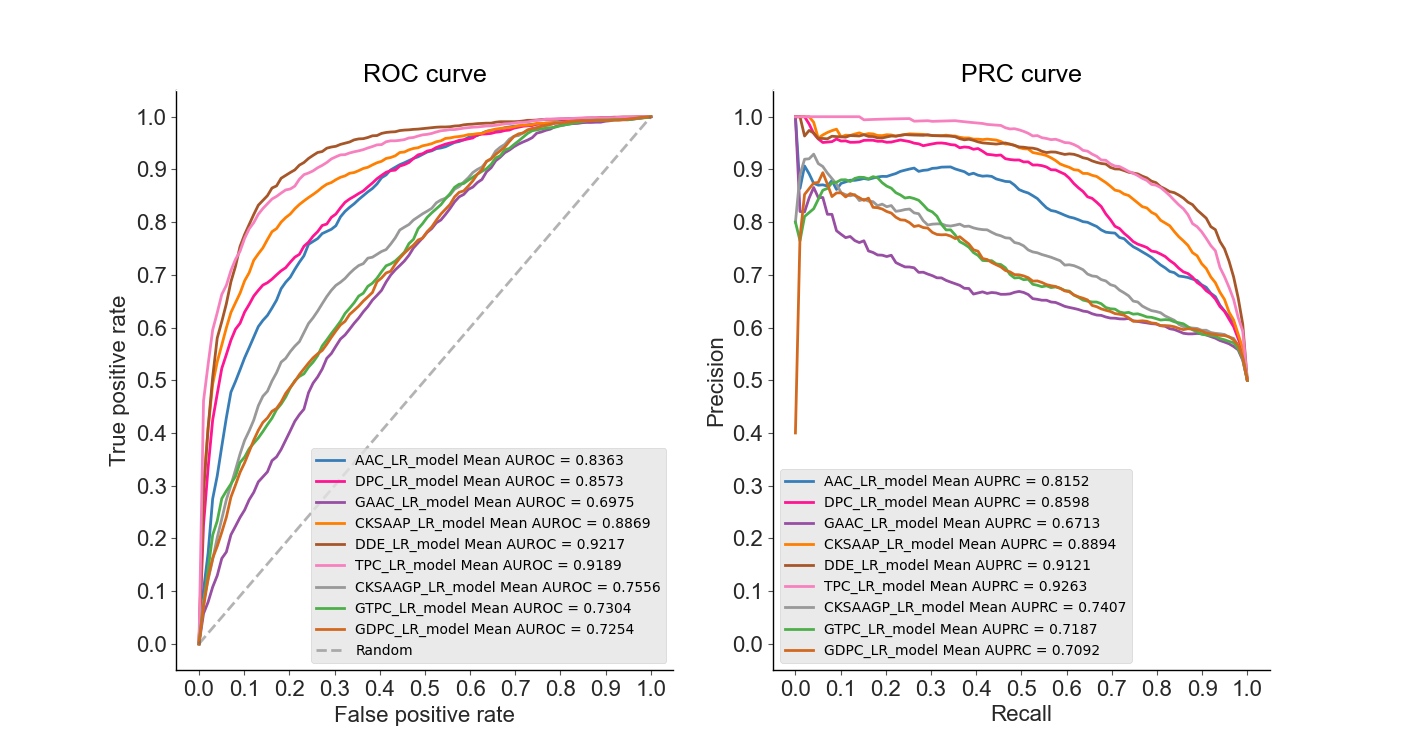
Supplementary Materials

**Supplementary Figure 1.** Performance of nine feature extraction methods based on LR algorithm.

**
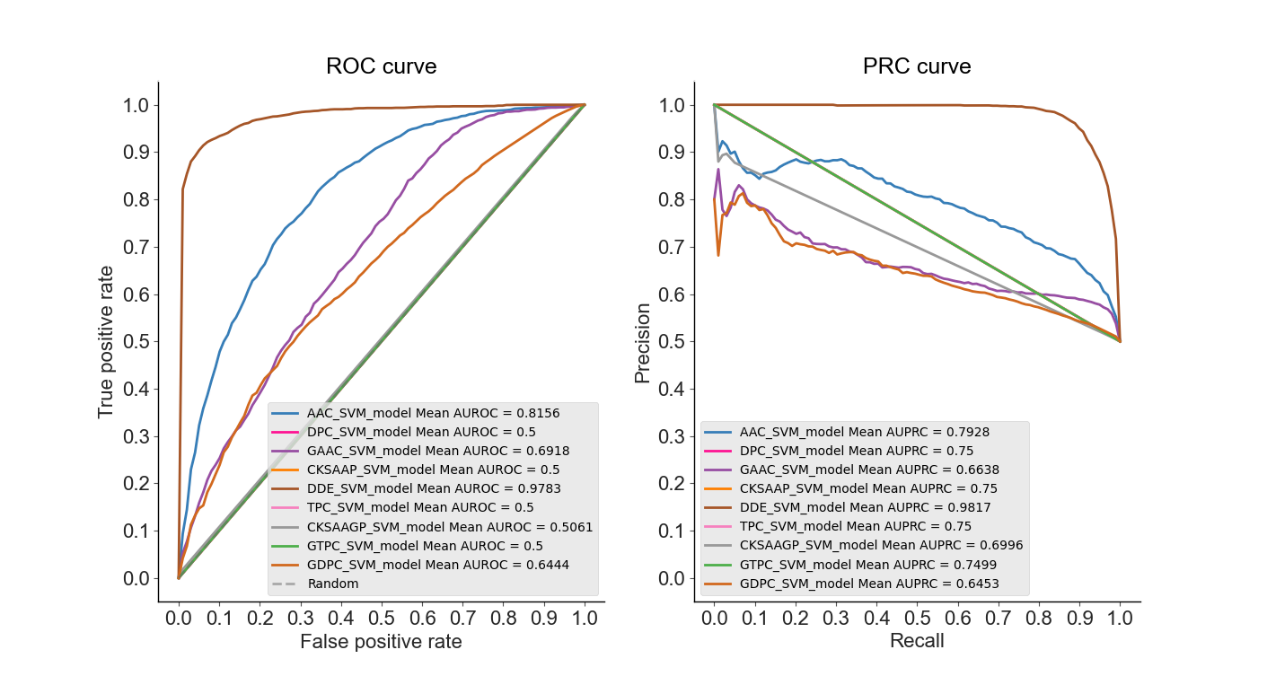
Supplementary Figure 2.** Performance of nine feature extraction methods based on SVM algorithm.

**
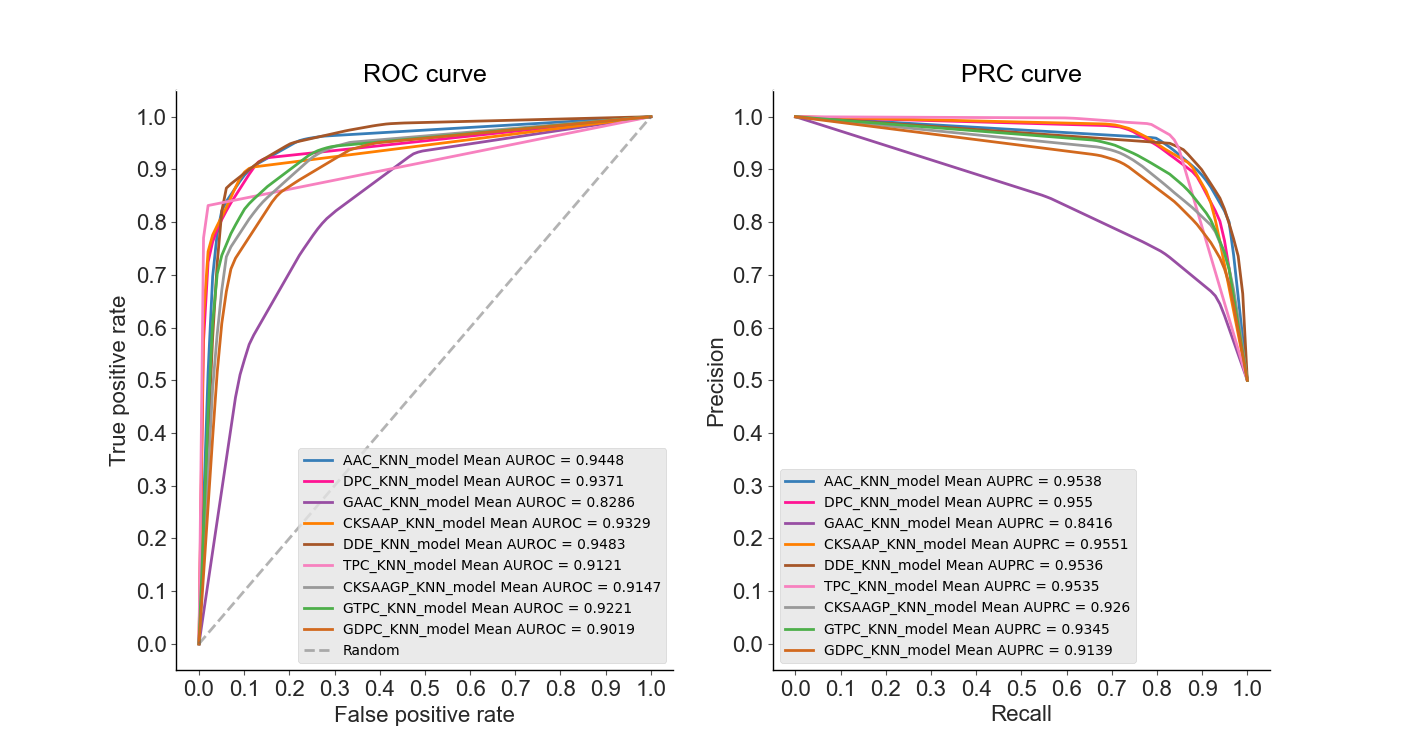
Supplementary Figure 3.** Performance of nine feature extraction methods based on KNN algorithm.

**Supplementary Table 1.** The detailed results of each fold *k*=1, 2, 3, 4, 5 for the optimal RF model testing on training dataset.

| *k* | *Sn* (%) | *Sp* (%) | *Pre (%)* | *Acc* (%) | *MCC* | *F1* |
| --- | --- | --- | --- | --- | --- | --- |
| 1 | 90.7 | 93.8 | 93.6 | 92.2 | 0.85 | 0.92 |
| 2 | 91.5 | 93.2 | 93.1 | 92.4 | 0.855 | 0.9 |
| 3 | 87.6 | 93.5 | 93.1 | 90.5 | 0.81 | 0.90 |
| 4 | 89.0 | 91.2 | 91.0 | 90.1 | 0.8 | 0.90 |
| 5 | 83.0 | 96.0 | 95.4 | 89.5 | 0.80 | 0.89 |
| Mean | 88.4 | 93.6 | 93.3 | 91.0 | 0.83 | 0.91 |
